# First administration to man of Org 25435, an intravenous anaesthetic: A Phase 1 Clinical Trial

**DOI:** 10.1186/1471-2253-10-10

**Published:** 2010-06-29

**Authors:** Ann E Rigby-Jones, J Robert Sneyd, Peter Vijn, Patrick Boen, Maurice Cross

**Affiliations:** 1 Peninsula College of Medicine & Dentistry, University of Plymouth, The John Bull Building, Research Way, Tamar Science Park, Plymouth PL6 8BU, UK; 2 Schering-Plough Corporation, 56 Livingston Ave., Roseland, NJ 07068, USA; 3 Veeda Clinical Research, 119 Looseleigh Lane, Derriford Plymouth PL6 5HH, UK

## Abstract

**Background:**

Org 25435 is a new water-soluble alpha-amino acid ester intravenous anaesthetic which proved satisfactory in animal studies. This study aimed to assess the safety, tolerability and efficacy of Org 25435 and to obtain preliminary pharmacodynamic and pharmacokinetic data.

**Methods:**

In the Short Infusion study 8 healthy male volunteers received a 1 minute infusion of 0.25, 0.5, 1.0, or 2.0 mg/kg (n = 2 per group); a further 10 received 3.0 mg/kg (n = 5) or 4.0 mg/kg (n = 5). Following preliminary pharmacokinetic modelling 7 subjects received a titrated 30 minute Target Controlled Infusion (TCI), total dose 5.8-20 mg/kg.

**Results:**

Within the Short Infusion study, all subjects were successfully anaesthetised at 3 and 4 mg/kg. Within the TCI study 5 subjects were anaesthetised and 2 showed signs of sedation. Org 25435 caused hypotension and tachycardia at doses over 2 mg/kg. Recovery from anaesthesia after a 30 min administration of Org 25435 was slow (13.7 min). Pharmacokinetic modelling suggests that the context sensitive half-time of Org 25435 is slightly shorter than that of propofol in infusions up to 20 minutes but progressively longer thereafter.

**Conclusions:**

Org 25435 is an effective intravenous anaesthetic in man at doses of 3 and 4 mg/kg given over 1 minute. Longer infusions can maintain anaesthesia but recovery is slow. Hypotension and tachycardia during anaesthesia and slow recovery of consciousness after cessation of drug administration suggest this compound has no advantages over currently available intravenous anaesthetics.

## Background

The GABA-a receptor mediates the effect of structurally diverse anaesthetics [1] and is a key target for intravenous hypnotics including etomidate, thiopentone, alphaxalone and propofol [2,3]. Current intravenous anaesthetics, such as propofol, are sparingly soluble in water and are therefore presented in complex formulations which may present problems including anaphylaxis and lipid accumulation. A water-soluble anaesthetic, minaxolone was developed in an attempt to avoid complex formulation issues but was ultimately withdrawn due to an unacceptable level of muscle movement and concerns over carcinogenicity.

**Figure 1:**
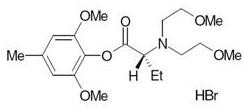
**Structure of Org 25435 [2,6-dimethoxy-4-methylphenyl-2'-N-bis(2'-methoxyethyl) aminobutyrate HBr]**.

In an attempt to develop a water-soluble alternative to propofol, a series of alpha-amino acid esters were synthesised from which Org 25435 ([2,6-dimethoxy-4-methylphenyl-2'-N-bis(2'-methoxyethyl) aminobutyrate HBr], Figure 1) was selected for clinical studies. Extensive pre-clinical evaluation showed it to be a potent intravenous anaesthetic with sleep times in mice comparable to propofol and a therapeutic index of 12 [4]. Org 25435 is an allosteric modulator of the GABA-a receptor and inhibited binding of [35S]t-butylbicyclophosphorothionate to the GABA-a receptor channel complex and potentiated the effects of GABA in oocytes expressing a1b3g2 containing receptors with a potency comparable to that of propofol in the same assay [4].

Additional animal studies were performed in mice, rats, dogs, and marmoset monkeys [4,5]. In these studies, Org 25435 was shown to induce and maintain anaesthesia with rapid offset when drug administration was discontinued [6]. Org 25435 exhibited an acceptable behavioural and cardiovascular profile, such that administration of Org 25435 to healthy human volunteers could be justified. Based on the data from the animal studies the anticipated human dose was approximately 1 to 4 mg/kg. The objectives of this study were to assess the safety, tolerability & efficacy of Org 25435 as an intravenous anaesthetic in healthy young male volunteers following a single intravenous infusion of 1-30 minutes and to determine preliminary pharmacodynamic and pharmacokinetic characteristics of the compound.

## Methods

The protocol and subsequent amendments were reviewed and approved by the Independent Ethics Committee (Plymouth) (Non-NHS Phase 1). All subjects gave written consent to participation. Clinical Trial Exemption Certificate, CTX, was not required for this study. The clinical trials registration number is NCT01062867.

### Setting and study subjects

The study was conducted in a specialist clinical trials unit with standard equipment for induction and maintenance of anaesthesia and subsequent supervised recovery. We studied healthy male subjects who were non-smokers and consumed less than 20 units of alcohol ( < 160 g) per week. All subjects underwent standard medical screening including laboratory investigations within 3 weeks of scheduled dosing.

### Study drug

Org 25435 was supplied as a freeze-dried cake stored at 2-8°C and reconstituted with water to 20 mg/ml.

### Short Infusion study

This was a dose escalation study. Dosing began at 0.25 mg/kg infused over one minute and increased to 0.5, 1.0, 2.0, 3.0, 4.0, 5.0 and 6.0 mg/kg in subsequent subjects. Beginning with the lowest dose, two subjects were studied at each dose. Once general anaesthesia occurred in any volunteer, the number of subjects studied at that dose and upwards increased to five. The study proceeded to one dose level above the first level at which three or more of the five subjects experienced general anaesthesia. Anaesthesia was defined as loss of speech contact, loss of eyelash reflex and dropping of a water-filled syringe. Where there was ambiguity or doubt, the decision as to whether or not anaesthesia occurred was taken by the senior anaesthetist present. Video recordings were available for review if necessary. Recovery endpoints included time of eye-opening on command and time of limb movement on command.

### Target Controlled Infusion (TCI)

A TCI study was performed after completion of the Short Infusion study and appropriate evaluations of safety. An interim evaluation of the pharmacokinetic data of the subjects treated in the Short Infusion study was performed to provide the pharmacokinetic parameter estimates which were used to control the infusion rate of the pump during the TCI. The TCI was given by a Harvard 22 pump controlled by STANPUMP software running on a personal computer. The 30 minute TCI aimed to achieve stable arterial plasma concentrations providing for a stable period of anaesthesia. Stable anaesthesia was defined as anaesthesia lasting for a consecutive period of 10 minutes or more.

### TCI infusion scheme

The 30 minute TCI was divided into two sections: 0-15 minutes and 15-30 minutes.

#### First TCI subject

The initial target concentration was based on the mean Org 25435 concentration occurring at the time at which eyes were opened on command in the short infusion study and evaluated using simulation to ensure that the total dose received (including any upwards titration), did not exceed 1.5 times the maximum dose given in the Short Infusion study.

If anaesthesia was achieved and maintained during the 0-15 minute section, the target concentration was to be maintained for the 15-30 min section; otherwise, after the first 0-15 minute section, the target concentration was to be increased by 50% for the remaining 15-30 min section.

#### Subsequent TCI subjects

The target concentration for the 0-15 minute infusion interval was to be set to the target concentration of the previous volunteer during the 15-30 minute infusion interval. However, if anaesthesia was *always *observed from the first volunteer onwards during the 0-15 minute infusion interval, the TCI target concentration during the 0-15 minute infusion interval for the next volunteer was to be set to 2/3 times the TCI target concentration of the previous volunteer during the 15-30 minute infusion interval.

Once stable anaesthesia was identified using the above rules in two volunteers at the same target concentration over the complete period of 30 minutes, the remaining volunteers to a maximum of seven were to be studied at target concentrations not more than 1.5 times that at which previous volunteers were studied. Dose escalation proceeded only after evaluation of safety and efficacy including review of all clinical and laboratory data (haematology and biochemistry) the response of subjects to dosing and comments from attending investigators.

The performance of the TCI model was assessed retrospectively by calculation of the bias, accuracy, divergence and wobble [7].

#### Pre-study preparations

Hartmann's Solution was infused intravenously (3 ml kg^-1^ hr^-1^) from at least 3 hours prior to the anticipated time of drug administration to ensure adequate hydration whilst fasting. An arterial cannula was inserted and a second venous cannula placed in an ante-cubital vein for infusion of Org 25435. Arterial blood samples (5 mL) were collected at 0, 1, 1.5, 2, 3, 4, 5, 8, 12, 16, 20, 45, 60, 120, 180, and 240 minutes after the start of infusion in the short infusion study and at 0, 3, 7, 11, 15, 18, 22, 26, 30, 30.5, 31, 32, 34, 37, 41, 45, 49, 59, 74, 89, 149, 209, and 269 minutes in the TCI study. The sample at 15 minutes was taken just prior to the potential alteration in target concentration. The sample at 30 minutes was taken just prior to stopping the infusion. A sample was also collected at the time eyes were opened to command.

Venous blood samples (5 mL) were collected at 60, 120, 180, 240, 360, and 720 minutes in the short infusion study and at 389, 509, and 749 minutes in the TCI study. Blood samples were processed to serum and stored at 20°C until analysis.

Urine was collected for 24 hours from subjects participating in the 4 mg/kg dose group of the Short Infusion study and in the TCI study.

After a stabilization period, during which baseline measurements were obtained, the anaesthetic infusion was started without notice. The times of eye closure, dropping water filled syringe, loss of eyelash reflex, eye opening on command and limb movement on command were recorded.

For safety reasons adverse events were recorded throughout the study.

### Electroencephalogram

Frontal electroencephalogram (differential recordings between FP1, FP2 locations international 10/20 system) was recorded from all volunteers using custom built equipment. Absolute power in two frequency bands, delta power (0.5-3.0 Hz) and theta power (3.25-8.0 Hz) were continuously calculated in subsequent epochs of 4 s.

### Quantification of Org 25435

Org 25435 and its internal standard, Org 24446, were isolated from serum using solid-phase extraction. Briefly, 10 ng Org 24446 was added to each 0.5 mL serum sample and the sample was vortex mixed for 3 seconds. The solid phase extraction cartridge (C18 (end-capped), 50 mg, 1 mL (ICT)) was conditioned with 1 mL methanol, followed by 1 mL water and then the serum sample was applied to the cartridge and allowed to run through under vacuum. The cartridge was then washed twice with 1 mL water. Org 25435 and Org 24446 were eluted from the cartridge with 1 mL acetonitrile.

The extracted samples were quantified by liquid chromatography (LiChroCART^® ^55-2 Purospher^® ^STAR RP-18 endcapped (3 μm)), coupled to a mass spectrometer (API 3000, Applied Biosystems) using turbo ion spray in multi reaction monitoring (MRM) mode, operating at 350°C and 5000 V. The mobile phase was run isocratically at a flow rate of 250 μL/min and consisted of 80% acetonitrile, 20% 0.01 mol/L ammonium acetate solution, pH = 4.2. The column oven temperature was set to 40°C. Operating conditions for the API 3000 were as follows: nebuliser gas 13, collision gas 5, turbo heater gas 8 L/min. The protonated M + H ^+ ^peak of Org 25435 (m/z 369.9) was used as the precursor ion and in the MRM-mode m/z 174.2 was measured as the product ion. The protonated M+H ^+ ^peak of Org 24446 (m/z 355.9) was used as the precursor ion and in the MRM-mode m/z 174.2 was measured as the product ion.

The lower limits of quantification of the assays were 5 ng/mL (serum) and 1 ng/mL (urine). The plasma assay was linear across the range 5 to 2000 ng/mL (r = 0.992). The plasma intra-assay coefficient of variation (CV) was less than 13% across the calibrated range, while the inter-assay CV was less than 15%. The urine assay was linear across the range 1 to 500 ng/mL (r = 0.997). The urine intra-assay coefficient of variation (CV) was less than 6% across the calibrated range, while the inter-assay CV was less than 12%.

### Pharmacokinetic modelling and simulation

Moment analysis was performed using standard techniques. Mixed-effects population models [8] were fitted to the Org25435 plasma concentration versus time data using the first order estimation method of the program NONMEM [9]. Arterial plasma concentrations were used preferentially (over venous concentrations) where available. When not available e.g. time points post 240 minutes (post 269 minutes in the TCI studies) after administration of the study drug, venous concentrations were used for analysis. Three-compartment kinetics were assumed for the interim model whereas three- and four-compartment models were compared for the final model. Models were parameterized in clearances and volumes. Parameters describing inter- individual variability (etas) were tested for their significance one at a time and were retained in the final population model if statistically justified. The relationship evaluated was as follows: P*i *= PTV*exp(eta*i*) where P*i *is the parameter value in the *i*th subject, PTV is the typical value of the parameter in the population, and eta*i *is a random variable with a mean of 0 and a variance of omega ^2^. Various residual errors models (proportional, additive and combined) were explored.

Two population models were produced:

1) An interim model describing Org25435 kinetics following short infusion. This was subsequently used for computer-controlled drug administration in the TCI study. The structural parameters (volume and clearances) for this model were assumed to vary according to volunteer body weight.

2) A final population model derived from Org25435 plasma concentration versus time data from both the short infusion and TCI studies. For the final population analysis which combined data from the short infusion study and the TCI study, the potential relationships between body weight and individual parameter estimates were explored.

The statistical significance of each proposed covariate-parameter relationship and the requirement for ETA parameters was assessed using the likelihood ratio test (where appropriate i.e. for nested models) and by consideration of the Akaike Information Criterion (non-nested models) and the precision of the final parameter estimates (all models). For nested models, the justification for each additional effect was for it to improve the goodness-of-fit statistic (-2 log likelihood) by more than 6.6 (evaluated against the chi-square distribution, this is equivalent to significance at the 0.01 level). The improvement (or lack of) in model goodness-of-fit was also assessed visually by the examination of diagnostic plots. After completing the model build, the necessity for each added component was re-assessed by removing it from the model and evaluating the resulting impact on the model fit. If the removed component caused an increase in the goodness-of-fit statistic equivalent to significance at the 0.005 level (i.e. the removal of the component significantly worsened the model), it was allowed to remain.

### Pharmacodynamic modelling

A sequential approach was taken to pharmacokinetic-pharmacodynamic modelling. That is, the final population pharmacokinetic model was used to derive individualised (post-hoc Bayesian) pharmacokinetic parameter estimates (e.g. rate constants and central compartment volume) for each volunteer in the short infusion groups. These were used as inputs, along with the drug dose and effect measure data, for the estimation of pharmacodynamic parameters. The absolute power in the theta band (3.25-8.0 Hz) was used as an effect measure and was described using a sigmoid E_max _model of Org 25435 concentration (CE) in an effect site compartment, according to the following equation:

where E_0 _is the baseline value, E_max _is the maximum response, EC _50 _is the effect site concentration corresponding to half of the maximum effect, and γ (Hill coefficient) describes the steepness of the drug concentration-effect relationship. The incorporation of a hypothetical effect compartment accounted for the hysteresis (temporal delay) between serum Org 25435 concentration and the onset of drug effect. This delay was characterised by the estimation of K_e0_, the blood-brain equilibration rate constant. NONMEM's first order conditional estimation method was used. Random residual error was described using an additive model. A proportional variance model was used to describe the inter-individual variability in E_max_, EC_50 _and K_e0 _and E_0_.

## Results

Nineteen healthy male volunteers were included into the dose escalation study and an additional 7 volunteers participated in the TCI study. Volunteers were 27.7 ± 4.8 years, weight 75.2 ± 7.6 kg, Body Mass Index 23.6 ± 2.1 kg/m2. Data are means ± SD. Two subjects per group received Org 25435 at 0.25, 0.5, 1.0, or 2.0 mg/kg; 5 received 3.0 mg/kg and 5 received 4.0 mg/kg in the short infusion study. Subject 10 (scheduled to receive 3 mg/kg) was excluded as the infusion was immediately terminated due to a leak. This subject was not included in any analysis except for safety.

### Induction and maintenance of anaesthesia

During the Short Infusion study, at 2 mg/kg both subjects were unresponsive but did not drop the syringe. All subjects were successfully anaesthetised at doses of 3.0 and 4.0 mg/kg.

**Figure 2:**
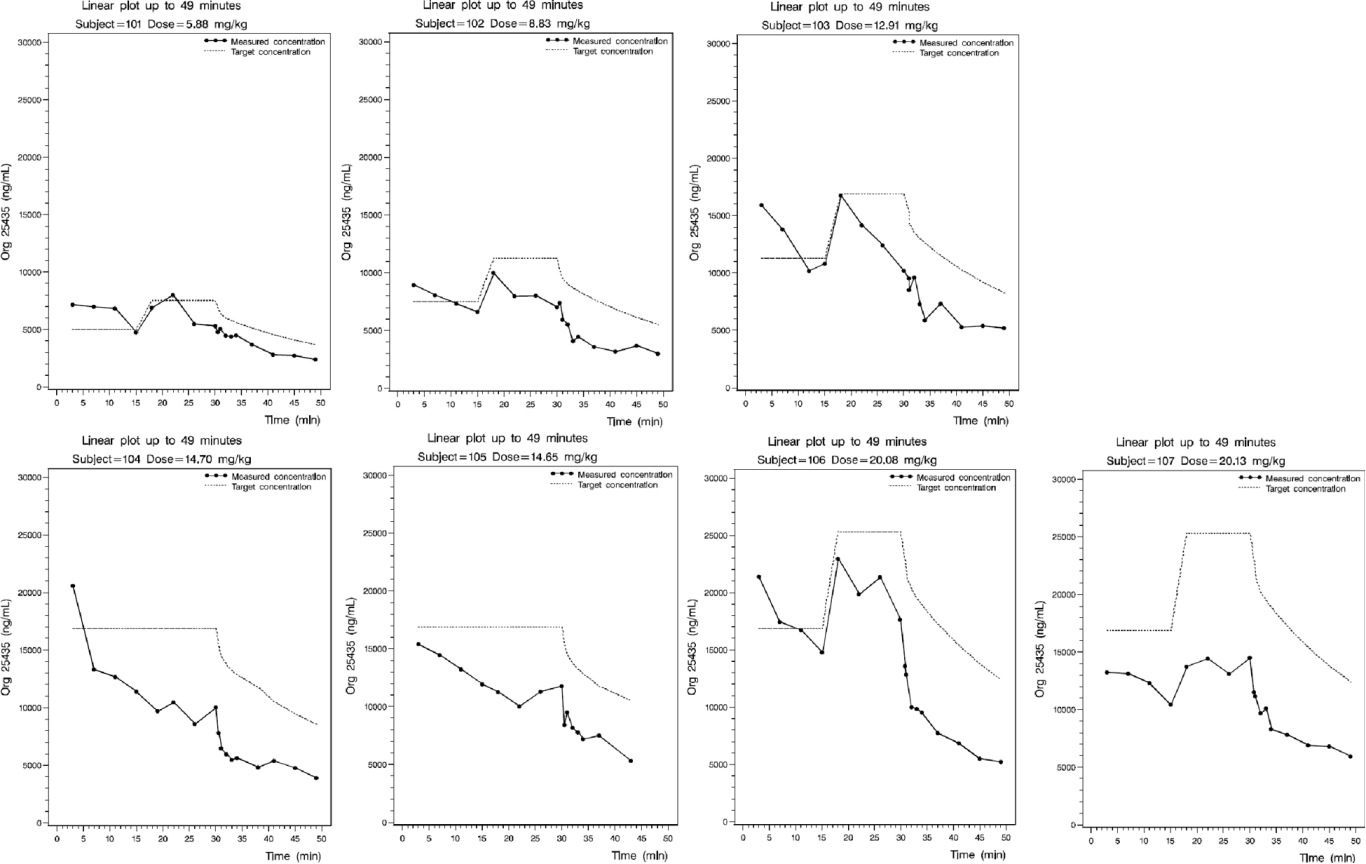
**Target and measured Org25435 plasma concentrations for each volunteer during the two 15 minute stages of the 30 minute infusion**. NB For volunteers 4 and 5, the target concentration remained the same (16.9ï¿½g/mL) for the entire 30 minute period.

Based on the mean Org 25435 concentration occurring at the time at which eyes were opened on command, a concentration of 5000 ng/mL was chosen as the target concentration for the first subject in the TCI study. Taking into account that the TCI target concentration during the 15-30 minute infusion interval was to be increased by 50% if stable anaesthesia was not observed in the 0-15 minute infusion interval, i.e. 7500 ng/mL, the maximum total amount infused in the first subject would be less than 6 mg/kg. Following interim pharmacokinetic modelling, 7 subjects received a titrated 30 minute Target Controlled Infusion (TCI). Total doses administered were: 5.8, 8.9, 12.9, 12.7, 14.8, 20.0 and 20.0 mg/kg. Figure 2 shows the target and measured plasma concentrations for each subject during the 30 minute infusion. Within the TCI study two subjects were observed to show periods of sedation, which lightened towards the end of the infusion, the other five subjects were successfully anaesthetised. Six out of 7 TCI subjects showed some evidence of twitching or other involuntary movements. The 5 anaesthetised subjects opened their eyes on command at 13.7 minutes and moved their limbs on command at 16 minutes after the TCI had finished, data are means.

The median absolute performance error (MDAPE) of the TCI pharmacokinetic model was 35.5%, the median performance error (MDPE) was -35.5%. The median values for divergence and wobble were 0.66%/hour and 10.8% respectively.

### Org 25435 serum concentration at 'time eyes opened on command'

For subjects 9-19 belonging to dose groups 3 and 4 mg/kg an arterial blood sample was taken at the time at which the eyes were opened on command. The mean Org 25435 concentration at which eyes were opened on command was 4462 ng/mL (median 4679 ng/mL). Table 1 provides the times of occurrence of various markers of sedation and the associated predicted effect site concentration of Org25435 for subjects in the short infusion group.

**Table 1:** Infusion study: induction of anaesthesia and recovery times

	Median	Min	Max	**Mean effect site conc**^**n **^**[SD]**	n
**2 mg kg^-1 ^(n = 2)**	**Time in minutes**			**μg mL^-1^**	

Cessation of speech	0.9	0.8	0.9	6.2	2
Eye closure	0.9	0.8	0.9	6.2	2
Syringe drop					0
Loss of eyelash reflex	1.7	1.7	1.7	10.9	1
Eyes open on command	2.1	2	2.2	8.3	2
Limb movement on command	2.1	2	2.2	8.3	2
					
3 mg kg^-1 ^(n = 5)					
Cessation of speech	0.8	0.5	0.9	8.3 [2.5]	5
Eye closure	0.8	0.8	1.1	10.3 [1.6]	5
Syringe drop	0.9	0.8	1.3	10.5 [1.5]	3
Loss of eyelash reflex	1.1	0.9	1.3	11.8 [2.1]	5
Eyes open on command	3.3	3.3	5.3	5.9 [1.3]	5
Limb movement on command	4.1	3.3	6.4	5.4 [1.4]	5
					
4 mg kg^-1 ^(n = 5)					
Cessation of speech	0.7	0.5	0.8	8.0 [1.4]	5
Eye closure	0.7	0.7	0.8	9.5 [0.7]	5
Syringe drop	0.9	0.7	1.2	12.2 [2.0]	5
Loss of eyelash reflex	1.2	1	2	15.5 [2.6]	5
Eyes open on command	5.3	3.2	9.2	6.4 [1.1]	5
Limb movement on command	5.8	3.7	9.4	6.1 [0.9]	5

Mean effect site concentration is the averaged individualised model predicted value of Org25435 in the biophase at time for each event.

### Haemodynamics

**Figure 3:**
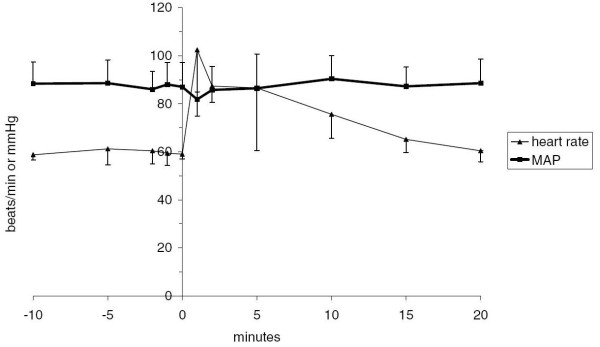
**Mean arterial blood pressures and heart rate before and after administration of 4 mg/kg Org 25435 over 1 minute, n = 5. The injection of Org 25435 caused an immediate tachycardia**.

Short infusions of Org 25435 at anaesthetic doses caused an immediate reduction in arterial blood pressure with an increase in heart rate. During TCI, there was a sustained reduction in arterial pressure and increase in heart rate and which gradually resolved after the infusions were discontinued. Systolic arterial pressure fell from 146 ± 13 to a minimum of 113 ± 14 mmHg at 37 min. The heart rate of subjects receiving TCI increased from 70 ± 6 beats min^-1 ^to a maximum of 102 ± 14 at 9 min. See Figure 3.

### Safety

There were 20 adverse events reported during the study including 6 reports of headaches and 2 cases of hot flushes. None of the adverse events were considered serious.

### Electroencephalogram

All electroencephalogram traces were inspected visually; no spiking or epileptiform activity was seen. Power increased during the Short Infusions of Org 25435 peaking before 2 minutes after the start of injection in most volunteers. Between 2 and 10 minutes, the effects washed out gradually. At the higher doses (3 and 4 mg/kg), after return of verbal contact, delta and theta power was still slightly higher than baseline, possibly indicating a residual light sedation.

### Pharmacokinetic analysis

Pharmacokinetic modelling was based on arterial plasma concentrations for the first four hours post-dose. Venous plasma concentrations were used for later time points i.e. 360 minutes onwards. The relative concentration difference, calculated as ((venous concentration-arterial concentration)/arterial concentration) in the 240 minute post-dose sample (latest sample for which both sample matrices were available) was always less than 1 ng/mL.

1) Short Infusion Study

**Figure 4:**
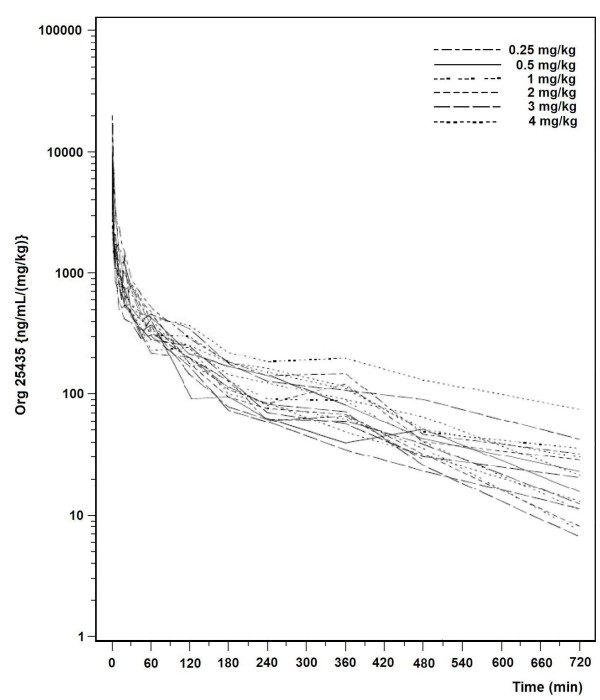
**Arterial concentrations of Org 25435 in 18 subjects receiving 0.25-4 mg/kg**. The measured concentrations have been normalized to a dose of 1 mg/kg.

Measured concentrations of Org 25435 from all subjects in the Short Infusion study are shown in Figure 4. Pharmacokinetic parameter estimates for the interim model, based on the Short Infusion study data only, are provided in Table 2.

**Table 2:** Pharmacokinetic parameter values for the interim model

Parameter	Typical Value	(SE)
CL mL kg^1 ^min^1^	7.99	(0.0241)
Q2 mL kg^1 ^min^1^	68.7	(2.97)
Q3 mL kg^1^min^1^	9.33	(0.506)
V1 mL/kg	57.7	(3.46)
V2 mL/kg	335	(9.90)
V3 mL/kg	993	(26.8)

Data are typical values (standard error). CL = irreversible systemic clearance from the central compartment; Q2 = distribution clearance for the rapidly equilibrating peripheral compartment; Q3 = distribution clearance for the slowly equilibrating peripheral compartment; V1= volume of the central compartment; V2 = volume of the rapid peripheral compartment; V3 = volume of the slow peripheral compartment.

2) Short Infusion & TCI Studies

#### Moment Analysis

Individual pharmacokinetic parameters calculated without assuming a specific compartment model are presented as Additional File 1. Plots of area-under-the-curve versus Org 25435 dose, for both short infusions and target controlled infusions, showed no evidence of non-linear pharmacokinetics (data not presented).

#### Urinary Excretion

0.005 ± 0.003% and 0.013 ± 0.001%, mean ± SD respectively of the administered dose was excreted unchanged between 0 and 24 hours.

#### Population modelling

##### Pharmacokinetics

**Figure 5:**
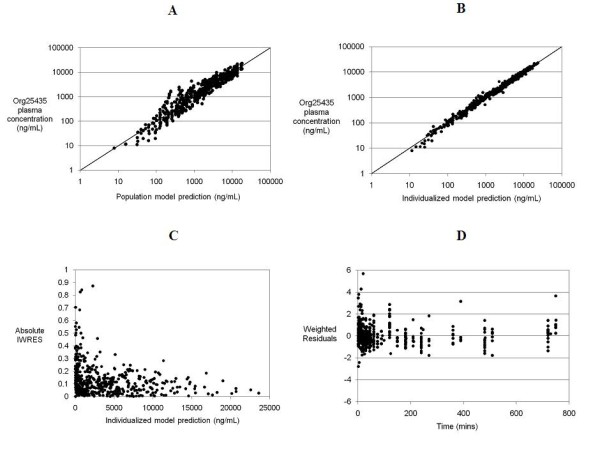
**Plots demonstrating the performance of the final pharmacokinetic model**. Panels A and B show the observed ORG25435 plasma concentration versus the population model predicted and individualized model predictions, respectively, demonstrating the overall model goodness-of-fit. Panel C shows the absolute values of the weighted residuals (approximating to standard deviation units) for the individualized model (IWRES) versus the individualized predictions. Panel D shows the weighted residuals (WRES) versus time.

As a first step three- and four-compartment mamillary models were compared. The four-compartment model described the data significantly better than the three-compartment model. The influence of body weight on the pharmacokinetic parameter estimates was then systematically examined. A four-compartment model, in which the structural parameters (volumes and clearances) increased in proportion to the individual's body weight, performed better than a model in which CL was not weight-normalised. Final model pharmacokinetic parameter estimates are shown in Table 3. Goodness-of-fit plots for this model are shown as Figure 5.

**Table 3:** Org 25435 pharmacokinetic parameter estimates. The model is based on 18 short (1 minute) infusions and 7 Target Controlled Infusions (30 minutes)

Parameter	Typical Value	95% Confidence Interval	CV%
CL mL kg^-1 ^min^-1^	8.31	7.23-9.39	31.3
Q2 mL kg^-1 ^min^-1^	13.7	-18.2* -45.6	
Q3 mL kg^-1 ^min^-1^	62.8	30.3-95.3	61.4
Q4 mL kg^-1 ^min^-1^	12.4	7.47-17.3	35.4
V1 mL kg^-1 ^min^-1^	38.7	18.9 - 58.5	
V2 mL kg^-1 ^min^-1^	56.2	-65.8* - 178	
V3 mL kg^-1 ^min^-1^	642	393 - 891	68.5
V4 mL kg^-1 ^min^-1^	1380	1137 - 1624	8.5
Residual variability %	20.3	13.9 - 25.1	

Confidence intervals were calculated as the typical parameter estimate ± 1.96 x standard error of the estimate. The coefficient of variation (CV%) was calculated as the square root of the variance of the eta variable associated with that parameter. CL = irreversible systemic clearance from the central compartment; Q2 = distribution clearance for the rapidly equilibrating peripheral compartment; Q3 = distribution clearance for the slower equilibrating peripheral compartment; Q4 = distribution clearance for the slowest equilibrating peripheral compartment; V1= volume of the central compartment; V2 = volume of the rapid peripheral compartment; V3 = volume of the slower peripheral compartment; V4 = volume of the slowest peripheral compartment. * These confidence intervals are symmetric approximations, the true confidence interval does not contain zero.

#### Pharmacodynamic modelling

**Figure 6:**
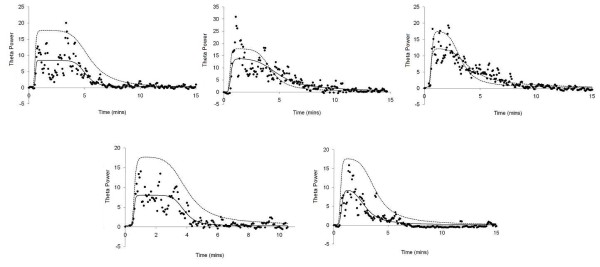
**Observed (marker), population model predicted (dashed line) and individualised model predicted (solid line) values of absolute theta power in the volunteers receiving Org 25435 at 4 mg kg ^-1^**.

Pharmacodynamic modelling was performed using the electroencephalogram data (absolute power in theta band) from the fourteen volunteers in the short infusion group (dose range 1 to 4 mg kg^-1^). Parameter values and plots of observed and model predicted values for theta for the volunteers who received 4 mg kg^-1^, are given in Table 4 and Figure 6, respectively.

**Table 4:** Pharmacodynamic parameters

Parameter	Typical Value	95% Confidence Interval	CV%
E_0_	0.158	-0.02 - 0.336	114
E_max_	17.8	15.6 - 19.9	93.3
EC_50 _ng/mL	7580	6699 - 8461	22.2
Gamma	6.28	5.03 - 7.53	35.6
K_e0_(min^-1^)	0.808	0.746 - 0.870	12.6
K_e0 _half life (min)	0.858	0.797 - 0.929	
Residual error	1.98	1.55 - 2.34	

Confidence intervals were calculated as the typical parameter (geometric mean) estimate ± 1.96 x standard error of the estimate. The coefficient of variation (CV%) was calculated, where possible, as the square root of the variance of the eta variable associated with that parameter.

## Discussion

The purpose of this study was to determine whether Org 25435 could induce anaesthesia in man and to evaluate safety in a limited number of subjects. The target controlled infusion (TCI) phase of the study explored the suitability of Org 25435 for maintenance of anaesthesia.

### Short Infusions

All subjects receiving the short (1 minute) infusions of 3 mg/kg or more became anaesthetised. This induction dose was within the predicted range from animal data.

The characteristics of the induction of anaesthesia were satisfactory. We were not able to definitively evaluate pain on injection because the drug was infused through an ante cubital vein. The sequence of induction "end points" also fitted with similar data from established intravenous agents. Specifically, the sequence was consistently loss of speech, then eye closure, then syringe drop, followed by loss of lash reflex.

For the minority of subjects (the 2 mg/kg group) who experienced sedation with a sub-anaesthetic dose of Org 25435, the experience was satisfactory. In those subjects who became anaesthetised (doses of 3 mg/kg and 4 mg/kg, n = 10) there was unequivocal anaesthesia which was clinically similar to that achieved with other agents. The small excitatory movements observed in 4 of the 10 subjects receiving 3 or 4 mg/kg did not compromise the safety of the subjects.

The increase in heart rate, and decrease in blood pressure, was consistent and substantial. Although our volunteers were healthy young males, similar changes induced in patients with cardiovascular instability or compromised myocardium would be a real clinical problem. Volunteer studies of a new intravenous formulation are potentially stressful for unpremedicated anxious subjects and it is possible that sympathetic activity was increased by factors unrelated to the study drug.

The electroencephalogram effects of Org 25435 are similar to those of other anaesthetics (synchronization of the electroencephalogram, shift in power towards lower frequencies) Time to maximum electroencephalogram effect after a Short Infusion was fast (mean predicted time to peak effect 1.42 minutes (SD 0.12), K _e0 _half life was 0.86 min, 95% CI 0.80 - 0.93) and the bulk of the EEG effects vanished within 4 to 8 minutes.

### Target controlled infusions

The TCI treatments induced anaesthesia in five subjects, the remaining two showing periods of sedation. The sedation/anaesthesia status of the subjects was not consistent during each of the plateaux and tended to lighten with the passage of time. This was confirmed by the measured arterial blood concentrations which tended to fall with time, presumably due to the inability of the preliminary pharmacokinetic model to adequately describe drug disposition during longer infusions. The modest excitatory phenomena seen during the short infusions, and subsequent anaesthesia appeared more often and to a greater magnitude in the subjects in whom anaesthesia was maintained. Not all the excitatory phenomena were comparable to those routinely seen with propofol [10]. Specifically some were greater including whole arm movements. The interpretation of these is somewhat complicated because the drug concentration was (in retrospect) known to be changing. The positive value derived for model divergence (0.66%/hour) indicates that the difference between predicted and actual Org25435 plasma concentrations increased over time. It is possible that the depth of anaesthesia progressively lightened and may have rendered the subjects into a condition more likely to develop excitation.

Recovery from TCI was slow. The relatively fast recovery from bolus/short infusions suggests that rapid distribution into peripheral compartments terminates the drug effect. However, the equilibration between compartments which takes place during a longer infusion exposes the importance of drug elimination. The value for metabolic clearance of Org25435 derived in this study, at 8.2 mL/kg/min, was approximately half the predicted value obtained from allometric scaling of pre-clinical pharmacokinetic data in rats and may partially explain the slower than anticipated recovery observed in this study after 30 minute infusions. It is well known that animals are imperfect predictors of drug handling in humans. Drugs may be metabolised in animals via pathways that are minor or absent in humans, leading to a slower than expected clearance. Certainly, there appears to be a meaningful clinical "problem" with recovery from longer infusions of Org25435 which was not anticipated from the preclinical studies [4]. Failure to predict slow recovery from 30 min infusions in man may reflect the limitations of extrapolating from brief infusions in small animal species. Successful extrapolation from rats to humans has been made for thiopental but the underpinning modelling was based on extensive prior knowledge of tissue kinetics [11].

### Pharmacokinetics

A negligible percentage of the Org 25435 dose administered was excreted in the urine unchanged, indicating that metabolism is the major mechanism for elimination of the compound. Currently, little is known about the metabolic products of Org25435 in man.

The moment (non-compartmental) and population pharmacokinetic analysis revealed no evidence of non-linear pharmacokinetics. However, the interim pharmacokinetic model developed from the short bolus infusion data failed to adequately predict the Org 25435 concentrations during controlled infusion. Fits of the final pharmacokinetic model developed using the concentration data from both the short bolus infusions as well as TCI, show that the final model does predict a fall in concentrations during TCI. In retrospect, moving to 30 minute TCI using a model derived from 1 min infusions was over-ambitious and the resulting non-stable anaesthesia was experimentally unsatisfactory. Future studies might usefully start with 10 min zero-order infusions to clearly define the hysteresis between arterial concentrations and brain effect and then move to shorter infusions to replicate clinical practise at induction and longer fixed rate infusions before attempting TCI.

**Figure 7:**
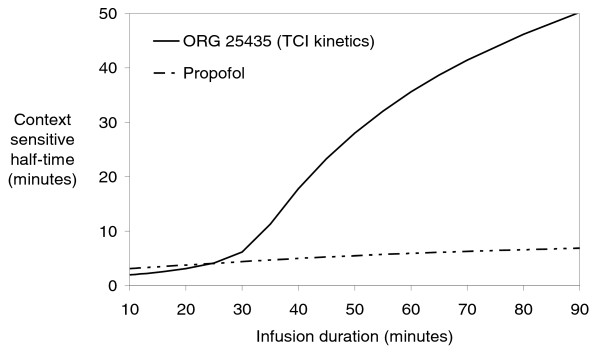
**Context sensitive half-time profiles for propofol and Org 25435 (kinetics based on TCI data only)**. Context sensitive half-time is defined as the time required for the plasma drug concentration to decline by 50% after termination of a drug infusion. The 'context' refers to the infusion duration.

The context-sensitive half-time has been proposed as a useful descriptor of post-infusion central compartment kinetics, and in connection with that, of recovery from drug effect [12,13]. Using a preliminary 3-compartment pharmacokinetic model based on the TCI data only, context-sensitive half-times of Org 25435 were predicted for infusions of varying duration. The context-sensitive half-time, although initially short (2 minutes) after a 10-minute infusion, increases progressively with increasing duration of infusion, reaching a value of 50 minutes after a 90 minute infusion. For comparison the predicted value for propofol was approximately 7 minutes [14], see Figure 7. Based on the concept of context sensitive half time, times to recovery from anaesthesia are expected to increase with increasing duration of Org 25435 infusion. This fits in with the prolonged time to recovery observed after 30 minute TCI compared to the short bolus infusions. However, extrapolation beyond 30 minutes is to go beyond the underpinning data and is, therefore speculative.

### Pharmacodynamic

We elected to collect raw EEG rather than use a simplified derived index of anaesthetic depth. This approach allowed us to look carefully for excitatory cortical phenomena whilst also providing a continuous measure of drug effect. Our modelling demonstrates that Org 25435 enjoys relatively rapid access to the effect site, with an estimated K_eO _half-life of 0.9 minutes. This contributes to the prompt return of consciousness after bolus injections but is outweighed by tissue accumulation which accounts for the slow recovery following extended infusion.

It was crucial for the correct characterisation of pharmacodynamics that our initial blood samples were arterial. In their studies of thiopental disposition, Homer and Stanski were only able to characterise K_eO _in patients from whom arterial, rather than venous, samples were collected [15]. Ideally, arterial samples would be collected for the entire duration of a pharmacokinetic study; however this is rarely achievable in clinical practice. Our approach of continuing to collect venous samples when arterial samples were no longer available, and thus accepting small arterio-venous differences beyond four hours, allowed full characterisation of the compound's elimination half-life and volume of distribution. To have ceased sampling at the time of arterial line removal would likely have greatly biased our data as the estimation of pharmacokinetic parameters is known to correlate with duration of sampling time [16]. For our data, at 240 minutes the arterial sample concentration was only 12.2 +/- 10.5% lower than the venous concentration and this difference would likely have been even smaller at 360 minutes, the time at which the first venous data entered the pharmacokinetic modelling process.

## Conclusions

Org 25435 is an effective intravenous anaesthetic in man at doses of 3 and 4 mg/kg, when administered over 1 minute. The adverse event profile of Org 25435 is unremarkable. No safety problems were seen with this preparation except for some cardiovascular phenomena and mild involuntary movements. Longer infusions can maintain anaesthesia but subsequent recovery is slow. Hypotension tachycardia during anaesthesia and slow recovery of consciousness after cessation of drug administration suggest this compound has no advantages over currently available intravenous anaesthetics.

## Competing interests

This study was funded by the manufacturers of Org25435, Organon Ltd, Oss, The Netherlands. All authors except Dr Rigby-Jones were employed by Organon Ltd or Veeda for their work in relation to this study.

## Authors' contributions

JRS participated in the study's design, coordination and clinical work. He also drafted the manuscript. AERJ developed the population pharmacokinetic and pharmacodynamic models and helped to draft the manuscript. PV led EEG analysis. PB participated in the study's design and coordination. MC participated in the study's design, coordination and clinical work. All authors read and approved the manuscript.

## Pre-publication history

The pre-publication history for this paper can be accessed here:

http://www.biomedcentral.com/1471-2253/10/10/prepub

## Supplementary Material

Additional file 1 **Results of moment analysis**. This file contains a summary table of non-compartmental pharmacokinetic parameter values (Means (SD)). Click here for file
