# Supplementary material for: First administration to man of Org 25435, an intravenous anaesthetic: A Phase 1 Clinical Trial
Source: BMC Anesthesiol. 2010 Jun 29;10:10. doi: 10.1186/1471-2253-10-10 (PMC2914736; doi:10.1186/1471-2253-10-10)
Supplement: Additional file 1 — Results of moment analysis. This file contains a summary table of non-compartmental pharmacokinetic parameter values (Means (SD)). [file 1471-2253-10-10-S1.DOCX]

# Additional files

### Additional file 1 – Results of moment analysis

|  |  | **Short infusion (mg/kg)** | | | | | | **TCI** |
| --- | --- | --- | --- | --- | --- | --- | --- | --- |
|  |  | **0.25** | **0.5** | **1.0** | **2.0** | **3.0** | **4.0** |  |
| **Parameter** | **[unit]** | **n=2** | **n=2** | **n=2** | **n=2** | **n=5** | **n=5** | **n=7** |
| dn−AUC_0−∞_ | [ng·min·mL^-1^·mg^-1^] | 2383 (387) | 1656 (296) | 1699 (6.78) | 1423 (71.2) | 1421 (289) | 1831 (639) | 1429 (700) |
| wn−CL | [mL·kg^-1^·min^-1^] | 6.16 (1.57) | 8.50 (1.53) | 8.33 (0.681) | 8.51 (1.20) | 10.1 (2.35) | 7.93 (1.92) | 10.1 (2.90) |
| wn−V_ss_ | [mL/kg] | 1368 (28.0) | 2645 (1035) | 1425 (69.8) | 2101 (728) | 2879 (942) | 2292 (408) | 3223 (743) |
| MRT | [min] | 129 (33.8) | 167 (18.8) | 126 (6.69) | 166 (57.8) | 188 (63.0) | 231 (67.2) | 231 (98.1) |
| t_½_ | [min] | 160 (43.9) | 211 (46.4) | 119 (3.90) | 177 (84.2) | 206 (73.0) | 205 (34.2) | 235 (61.9) |

Summary of Pharmacokinetic Parameters (Means (SD)) dn = dose-normalized; wn = weight-normalized.
